# Role of genetic polymorphisms in tumour angiogenesis

**DOI:** 10.1038/sj.bjc.6600625

**Published:** 2002-11-04

**Authors:** S P Balasubramanian, N J Brown, M W R Reed

**Affiliations:** Academic Unit of Surgical Oncology, University of Sheffield, Sheffield S10 2JF, UK; Academic Unit of Surgery, University of Sheffield, Sheffield S10 2JF, UK

**Keywords:** tumour angiogenesis, genetic polymorphism(s)

## Abstract

Angiogenesis plays a crucial role in the development, growth and spread of solid tumours. Pro- and anti-angiogenic factors are abnormally expressed in tumours, influencing tumour angiogenesis, growth and progression. Polymorphisms in genes encoding angiogenic factors or their receptors may alter protein expression and/or activity. This article reviews the literature to determine the possible role of angiogenesis-related polymorphisms in cancer. Further research studies in this potentially crucial area of tumour biology are proposed.

*British Journal of Cancer* (2002) **87**, 1057–1065. doi:10.1038/sj.bjc.6600625 www.bjcancer.com

© 2002 Cancer Research UK

## TUMOUR ANGIOGENESIS

Angiogenesis is a complex cascade of events involving extensive interplay between cells, soluble factors and extra-cellular matrix components. Soluble factors including cytokines have a stimulatory or inhibitory role, thereby regulating the process. The angiogenic potential of tumours was initially demonstrated in animal models and it is now recognised that angiogenesis not only precedes tumour growth, but is also necessary for metastasis. In the normal adult vasculature, a balance of the positive and negative angiogenic signals maintains quiescence. However, in the tumour microenvironment, angiogenesis occurs as there is either a preponderance of pro-angiogenic molecules or a decrease in anti-angiogenic stimuli.

## GENETIC POLYMORPHISMS IN ANGIOGENIC GENES AND RELEVANCE TO CANCER CARE

Polymorphisms are naturally occurring DNA sequence variations, which differ from gene mutations in that they occur in the ‘normal’ healthy population and have a frequency of at least 1%. Approximately 90% of DNA polymorphisms are single nucleotide polymorphisms (SNPs) due to single base substitutions. Others include insertion/deletion polymorphisms, minisatellite and microsatellite polymorphisms. Although most polymorphisms are functionally neutral, some have effects on regulation of gene expression or on the function of the coded protein. These functional polymorphisms, despite being of low penetrance, could contribute to the differences between individuals in susceptibility to and severity of disease. Certain polymorphisms alone, in combination or by interaction with environmental factors may affect the angiogenic pathway and thereby susceptibility and/or severity of cancers. Detection of the role of angiogenic gene polymorphisms that influence cancer susceptibility and/or severity may improve our understanding of tumour angiogenesis and may influence risk stratification and detection, use of new treatment strategies and prognostication of disease. The efficacy of anti-angiogenic treatment in solid cancer (Jain, 2001) could be further enhanced, if the individual angiogenic potential could be predicted on the basis of genotype.

The article reviews the role of polymorphisms in genes encoding factors and receptors that influence tumour angiogenesis. Whilst numerous polymorphisms have been identified, we have confined this review to those that are thought to be functionally important and may influence angiogenesis. Table 1

**Table 1:**
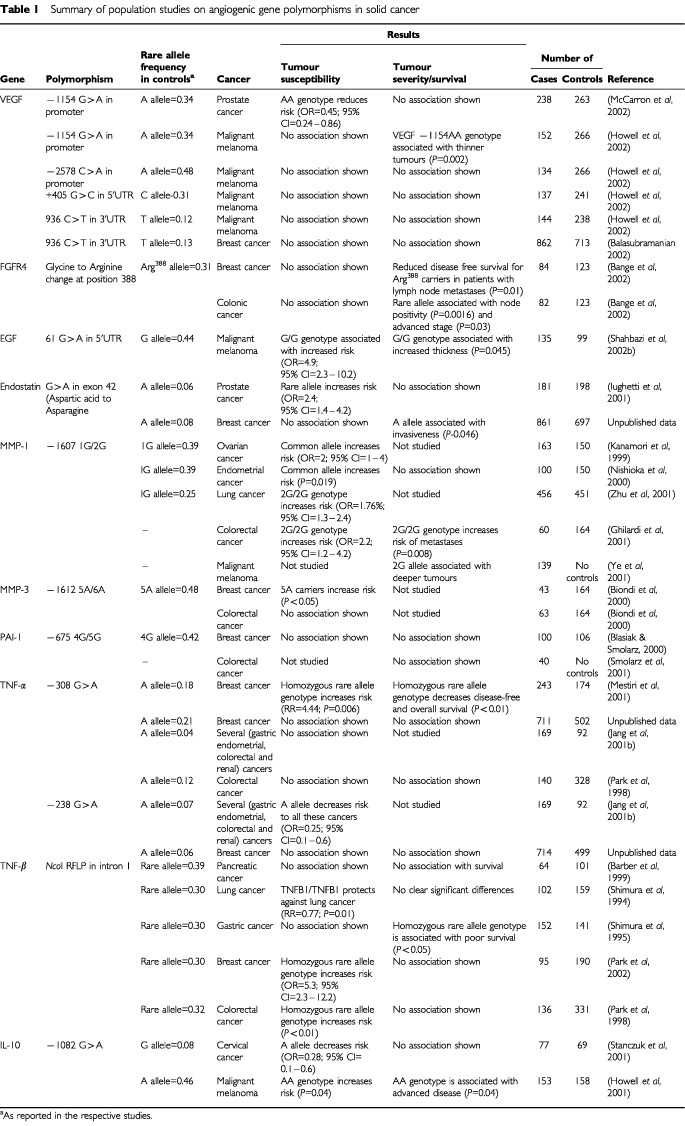
Summary of population studies on angiogenic gene polymorphisms in solid cancer

summarises the population studies that have evaluated a number of the genetic polymorphisms that will be discussed. Some ‘mutations’ with potential functional significance have been discussed briefly, as their prevalence in the normal population is as yet unknown. Factors/genes, which demonstrate minimal or indirect effects on angiogenesis such as tumour suppressor genes, oncogenes, hormones and hematopoietic factors, are not discussed in this review.

## VASCULAR ENDOTHELIAL GROWTH FACTOR

Vascular endothelial growth factor (VEGF or VEGF-A) is one of a family of six protein isoforms expressed in different tissues including brain, kidney, liver and spleen. VEGF stimulates proliferation, migration and tube formation of endothelial cells *in vitro* and regulates vascular permeability *in vivo* (Veikkola and Alitalo, 1999). VEGF expression correlates with angiogenesis and prognosis in several tumours including breast, lung and malignant mesothelioma (Toi *et al*, 2001; Strizzi *et al*, 2001).

Several polymorphisms have been described within the promoter and 5′UTR of the VEGF gene, some of which (+405 C>G, −1154G>A and –2578C>A) correlate with VEGF production. The +405C, –1154G and –2578C alleles are associated with low VEGF production (Watson *et al*, 2000; Shahbazi *et al*, 2002a). Recent studies have shown that in individuals with the –1154 AA genotype, there may be a decrease in prostate cancer risk (Mccarron *et al*, 2002) and reduction in invasive potential of malignant melanomas (Howell *et al*, 2002). This confirms earlier studies that associate reduced VEGF production with the −1154A allele.

In another study of healthy individuals, three polymorphisms (702 C>T, 936 C>T and 1612 G>A) were identified in the 3′UTR region of the VEGF gene. The 936 T allele was associated with decreased VEGF plasma levels (Renner *et al*, 2000). We have studied this polymorphism in breast cancer, but were unable to demonstrate any significant association (Balasubramanian *et al*, 2002).

## FIBROBLAST GROWTH FACTOR

The fibroblast growth factor (FGF) family includes 20 polypeptide growth factors sharing a central core of 140 amino acids (Powers *et al*, 2000). Four receptors (FGFR 1 to 4) belonging to the immunoglobulin super family have been identified. In addition to effects on inflammation, repair and tissue regeneration, FGFs also stimulate the proliferation and migration of endothelial cells, important in the process of angiogenesis. FGFs promote tumour cell mitosis and angiogenesis and inhibit apoptosis (Powers *et al*, 2000). FGFs, along with VEGF, play a major role in tumour angiogenesis and their inhibition represses tumour growth (Compagni *et al*, 2000). To date, no activating mutations have been documented in FGFs. However, disturbances in the regulation of FGF signalling can also occur at the receptor level. FGFRs are over expressed in many human tumours (Shingu *et al*, 1998; Yoshimura *et al*, 1998; Giri *et al*, 1999). Many point mutations of FGFRs, found in developmental defects such as dwarfisms and craniosynostotic syndromes, result in ligand-independent activation of the FGFRs (Neilson and Friesel, 1996). A description of all these mutations is beyond the scope of this review. However, similar mutations have also been identified in human gastric cancer (FGFR2 gene) and colorectal cancer (FGFR3 gene) (Jang *et al*, 2001a). A mutation in the activation loop of the FGFR3 kinase domain (Lysine to Glutamic acid change), leading to constitutive activation of FGFR3, has been identified in patients with multiple myeloma, bladder and cervical carcinomas (Hart *et al*, 2000). In the FGFR4 gene, a common polymorphism (G>A) exists in the coding region and results in an amino acid change (glycine to arginine change) in the transmembrane domain of the receptor. Recently, it has been shown that the FGFR4 Arg 388 allele may predispose breast and colonic cancer patients to rapid disease progression (Bange *et al*, 2002). Such genetic changes might therefore influence the process of carcinogenesis by affecting the intracellular signal transduction cascades following FGF stimulation. Table 2

**Table 2:**
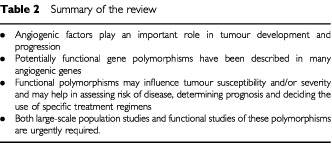
Summary of the review

## EPIDERMAL GROWTH FACTOR

Epidermal growth factor (EGF) exerts effects on cell proliferation and differentiation by binding to a tyrosine kinase receptor EGF receptor (EGFR). Although EGF can have direct effects on tumour cells, it also promotes angiogenesis, predominantly through a mitogenic effect on endothelial cells (Dunn *et al*, 2000). EGF, acting through its receptor also stimulates the production of vascular endothelial growth factor by tumour cells (Goldman *et al*, 1993), contributing further to the angiogenic response.

A polymorphism in the 5′ UTR of the EGF gene (61 G>A) appears to predispose to the development of malignant melanoma. The G allele is associated with higher *in vitro* EGF production (Shahbazi *et al*, 2002b).

## HEPATOCYTE GROWTH FACTOR/SCATTER FACTOR

Hepatocyte growth factor (scatter factor; HGF/SF), a heparin-binding glycoprotein binds to a tyrosine kinase receptor, which is the protein product of the c-met proto-oncogene. HGF-Met signalling is involved in developmental and homeostatic processes and also regulates neoplastic growth and progression (To and Tsao, 1998). It stimulates endothelial cell proliferation and migration and regulates vascular endothelial cell growth factor expression in vascular smooth muscle cells (van Belle *et al*, 1998). Increased Met and HGF/SF expression occurs in many tumours (To and Tsao, 1998) and the HGF/Met autocrine signalling pathway possibly has an oncogenic role (Takayama *et al*, 1997).

Somatic mutations of the c-*met* gene in patients with renal cell carcinoma have been shown to be oncogenic (Jeffers *et al*, 1997). Met is highly expressed in human gastric carcinoma cell lines, and a mutation (germ line missense Met mutation, P1009S) recently identified in gastric cancer displays increased and persistent tyrosine phosphorylation, when treated with HGF. Activating missense Met mutations could therefore contribute to gastric cancer tumorigenesis (Lee *et al*, 2000). Further investigations are required to determine the role of polymorphisms in the Met gene in angiogenesis and tumorigenesis.

## TRANSFORMING GROWTH FACTOR-BETA

Transforming growth factor-beta (TGF-β) is a 25-kDa protein which binds to three membrane molecules, TGF-β receptors type I, II and III (Koli and Keski-Oja, 1996). TGF-βs are potent regulators of cellular proliferation, differentiation and morphogenesis, as well as extra-cellular matrix formation, extra-cellular proteolysis, and inflammation. Although TGF-β inhibits cell proliferation, neoplastic cells acquire resistance to this inhibitory activity. TGF-β induces angiogenesis (Pertovaara *et al*, 1994) and this, together with effects on stromal formation and immune function, suggests involvement in tumour progression (Gregoire and Lieubeau, 1995). In non-small cell lung cancer, TGF-β1 protein level correlates with microvessel density and prognosis (Hasegawa *et al*, 2001).

Several polymorphisms have been identified in the TGF-β1 gene and have been studied in ischaemic heart disease (Andreotti *et al*, 2002). One coding polymorphism, (T>C resulting in Leucine to Proline change) has been associated with serum TGF-β1 levels; individuals with CC genotype having higher levels than TT or TC genotype (Yokota *et al*, 2000). Mutations have also been identified in the exons of TGF-β1 gene in breast tumour samples (Cardillo *et al*, 1997a), ovarian tumours (Cardillo *et al*, 1997b) and colorectal carcinomas (Cardillo and Yap, 1997). However, as yet no correlation has been identified between these mutations and mRNA and/or protein expression in tumours.

TGFBR2, a receptor for TGF-β lies close to or within one of the interstitial deletions that occur in 30–50% of head and neck, breast, and small cell lung cancers (Lucke *et al*, 2001). TGFBR2 gene mutations occur in colorectal and breast cancers and can result in absent receptor expression at the cell surface and defective TGF-β signalling pathways (Lucke *et al*, 2001). Potentially, these mutations may have a significant influence on tumour progression.

## ENDOSTATIN

Endostatin is a cleavage product of the COOH-terminal domain of collagen XVIII, which inhibits endothelial cell proliferation *in vitro* and tumour angiogenesis and growth *in vivo* (O'Reilly *et al*, 1997). Endostatin inhibits the growth of melanoma, fibrosarcoma, renal cell, mammary and ovarian cancer (Sim *et al*, 2000), and is currently being assessed in clinical trials.

Several polymorphisms have been described in the Endostatin gene (chromosome 21) (Iughetti *et al*, 2001). One polymorphism, (G>A in exon 42), which results in the change of aspartic acid to asparagine, is associated with prostate cancer susceptibility (Iughetti *et al*, 2001). In our study of breast cancer patients, the rare allele of this polymorphism appears to predispose to tumour invasion (unpublished data).

## MATRIX METALLOPROTEINASES

The matrix metalloproteinases (MMPs) are a family of highly conserved zinc-dependent endopeptidases with 20 members identified to date. Several MMPs have been localised to a chromosomal region (in 11q23), which is amplified in many solid tumours (Curran and Murray, 1999). In addition to regulating the growth of primary and secondary tumours, MMPs promote tumour angiogenesis and metastasis by extracellular matrix remodelling. Altered MMP expression occurs in tumours of the oesophagus, stomach, colorectal, pancreatic, breast, prostate, lung and ovary with levels correlating with disease stage and possibly prognosis (Curran and Murray, 1999). MMP activity is influenced by transcription regulation, latent MMP activation, and inhibition by endogenous tissue inhibitors of metalloproteinases (TIMPs) (Ye, 2000).

Polymorphisms in human MMP genes are associated with susceptibility and/or progression of various benign and malignant conditions. MMP-1, the most widely expressed metalloproteinase degrades interstitial collagens (types I, II and III), and over expression is implicated in tumour invasion and metastasis (Ye, 2000). An insertion/deletion polymorphism at position –1607 (1G/2G) results in increased binding (of the 2G allele) to recombinant Ets-1 transcription factor and increased transcriptional activity (Rutter *et al*, 1998). This allele is associated with increased MMP-1 expression in ovarian (Kanamori *et al*, 1999) and endometrial tumour samples (Nishioka *et al*, 2000) and found more frequently in ovarian cancer patients than in controls (Kanamori *et al*, 1999). Recent studies have shown that the 2G allele confers an increased susceptibility to lung cancer (Zhu *et al*, 2001) and predisposition to severe disease in colorectal tumours (Ghilardi *et al*, 2001) and malignant melanoma (Ye *et al*, 2001).

MMP-3 is also over-expressed in tumour tissues. In addition to extracellular matrix degradation, MMP3 activates gelatinase B and collagenases and releases cell surface molecules including E-cadherin, which in turn promote tumour growth (Biondi *et al*, 2000). A polymorphism in MMP-3 (5A/6A at position –1612 relative to the transcription track site) has been studied extensively in vascular disease. The 5A allele has increased promoter activity and decreased binding to an inhibitory transcription factor (Borghaei *et al*, 1999). This allele has been associated with breast cancer, but not with colorectal cancer (Biondi *et al*, 2000). Further studies are indicated due to the small size of the study.

MMP-9 (gelatinase B) is over-expressed in both breast and colorectal cancer (Garbett *et al*, 1999). Of the many identified polymorphisms in the MMP-9 gene, two (a (CA)n microsatellite polymorphism at position –90 and a single nucleotide polymorphism at position –1562) are functionally significant (Shimajiri *et al*, 1999; Zhang *et al*, 1999a). Studies have shown differences in the promoter activity between different (CA)n alleles (Shimajiri *et al*, 1999). The T allele of the C-1562T polymorphism demonstrates increased transcriptional activity (Ye, 2000) and is associated with severity of coronary atherosclerosis (Zhang *et al*, 1999b). Another polymorphism detected in MMP-12 (−82 A>G), also over-expressed in tumours, regulates MMP-12 expression (Jormsjo *et al*, 2000).

Tissue inhibitors of matrix metalloproteinases (TIMPs) specifically inhibit the active forms of the MMPs. There are four TIMPs (TIMP1–4) encoded by genes on different chromosomes (Apte *et al*, 1994). Recent research paradoxically suggests a positive correlation between TIMP levels and poor outcome (Curran and Murray, 1999). Three polymorphisms (−899T>A, −915A>G and –1296T>C) have been identified in the promoter region of the TIMP-3 (Beranek *et al*, 2000), although the functional significance is as yet unknown.

## PLASMINOGEN ACTIVATOR SYSTEM

Urokinase plasminogen activator (uPA) and tissue type plasminogen activator (tPA)), activators of the plasminogen system are countered by naturally occurring plasminogen activator inhibitors (PAI). uPA belongs to a family of serine proteases and is produced by both normal and tumour cells. It plays a major role in embryogenesis, ovulation, wound healing, inflammation, rheumatoid arthritis and in cancer growth, angiogenesis and metastasis (Hildenbrand *et al*, 1995; Rabbani, 1998). High levels of uPA, uPA receptor (uPAR) and paradoxically PAI-1 are associated with a poor prognosis in many human cancers (Nekarda *et al*, 1994; Pedersen *et al*, 1994; Foekens *et al*, 2000). This may be due to excess PAI-1 release facilitating re-implantation of circulating tumour cells, as stroma formation at the metastatic site requires the blockade of uPA-mediated degradation of the extracellular matrix (Janicke *et al*, 1991).

Two polymorphisms have been identified in the uPA gene (Conne *et al*, 1997). A polymorphic microsatellite marker exists in the uPAR gene, certain alleles of which have been found in colorectal cancer cell lines but not in healthy individuals (Kohonen-Corish *et al*, 1996). The PAI-1 gene has several polymorphic loci including a 3′ *Hind*III restriction fragment length polymorphism, a CA(n) dinucleotide repeat in intron 3 and a 4G/5G insertion/deletion at position –675 in the promoter. The smaller dinucleotide repeats are associated with higher plasma PAI-1 levels (Dawson *et al*, 1991) and the –675 4G allele demonstrates increased PAI-1 activity when compared to the 5G allele (Panahloo *et al*, 1995). Studies of the 4G/5G polymorphism in small numbers of breast (Blasiak and Smolarz, 2000) and colorectal cancer patients (Smolarz *et al*, 2001) have not revealed any association with cancer. Larger studies are required to establish a role for polymorphisms in the PAI-1 gene.

## CYTOKINES

In addition to specific angiogenic factors, certain cytokines are also involved in regulation of angiogenesis. Cytokine gene polymorphisms with a specific role in angiogenesis are therefore reviewed in the following sections.

### Tumour necrosis factor

#### TNF-α

Tumour necrosis factor (TNF)-α plays a critical role in the pathogenesis of various inflammatory, autoimmune and malignant diseases (Bazzoni and Beutler, 1996). Initially thought to have anti-tumour effects, TNF-α was later shown to be tumorigenic *in vivo*, with high plasma TNF-α levels associated with poor disease outcome (Warzocha *et al*, 1997). Stimulation of angiogenesis by TNF-α are well recognised, with effects modulated by other angiogenic factors (Yoshida *et al*, 1997).

Functional polymorphisms in the promoter region of the TNF-α gene at position –308 and –238 are associated with increased severity of infectious diseases, autoimmune diseases, and non-Hodgkin's lymphomas (Tsukasaki *et al*, 2001). The functional significance of several other TNF-α polymorphisms have recently been reviewed in detail (Hajeer and Hutchinson, 2001). The uncommon allele of the –308 polymorphism (TNF2) is associated with higher constitutive and inducible levels of TNF-α (Wilson *et al*, 1997). Individuals with the TNF2 homozygous genotype demonstrate an increased predisposition to breast cancer (RR, 4.44; *P*=0.006). In addition, the TNF2 homozygous genotype appears to be an independent prognostic indicator for both disease free survival and overall survival (Mestiri *et al*, 2001). However, this polymorphism does not influence colorectal cancer risk or severity (Park *et al*, 1998). The –238 A allele has been reported to be protective against cancers in general (Jang *et al*, 2001b), but this needs to be confirmed in larger studies. We have recently studied both the –308 and –238 polymorphisms in 711 breast cancer patients and 498 age and sex-matched controls, but were unable to demonstrate any association (unpublished data). Three additional polymorphisms located in the 5′-flanking promoter/enhancer region of the TNF-α gene at positions –1031(T>C), −863(C>A), and –857 (C>T) are associated with TNF-α production, all rare alleles being associated with higher levels (Higuchi *et al*, 1998). The TNF-α-857T allele is associated with adult T-cell leukaemia/lymphoma in the Japanese population (Tsukasaki *et al*, 2001). The effects on angiogenesis and the presence of many functional polymorphisms in the TNF-α gene certainly warrant further study in neoplastic diseases.

#### TNF-β (Lymphotoxin-α)

Structurally related to TNF-α, stimulates the production of VEGF in prostate cancer cell lines (Ferrer *et al*, 1998). Increased serum levels have been associated with progression in cervical cancer (Chopra *et al*, 1998). A *Nco*I RFLP exists in the first intron of TNFB (gene for TNF-β); the rare allele of which (TNFB*2) is associated with reduced TNF-β production (Messer *et al*, 1991). The homozygous common allele genotype (TNFB*1/TNFB*1) seems to protect against lung cancer (Shimura *et al*, 1994), colorectal cancer (Park *et al*, 1998) and breast cancer (Park *et al*, 2002). Studies on gastric cancer have also shown a prolonged survival in patients with this genotype (Shimura *et al*, 1995), whereas no association with either susceptibility or survival was demonstrated in pancreatic cancer (Barber *et al*, 1999).

### Interferons

Interferons (IFN) have a multitude of biological effects on growth and immunity, including modulation of gene expression, inhibition of viral replication, immunomodulation, decreased cell proliferation, suppression of oncogene expression and alterations in differentiation. There are three main types: Interferon-α, Interferon-β (or Type I Interferons) and Interferon-γ (Type II interferon). Several mechanisms, including inhibition of tumour angiogenesis, mediate the anti-tumour effects (Lindner and Borden, 1997). Interferon-α inhibits angiogenesis by down-regulating the expression of FGF-2 (Dinney *et al*, 1998). Interferon-β inhibit angiogenesis, possibly by enhanced IFN-induced gene expression and this effect is enhanced when combined with Tamoxifen (Lindner and Borden, 1997).

Several polymorphisms have been described in the interferon-α genes (Golovleva *et al*, 1996; Muldoon *et al*, 2001). However, as majority of the members of the IFN-α family have widely overlapping functions, mutations in any one of the several encoding genes may only result in minor functional consequences.

Interferon-γ induces expression of IFN-inducible protein 10 (IP-10), a potent inhibitor of angiogenesis and tumour growth *in vivo* (Sgadari *et al*, 1996).

Of the several polymorphisms described in the IFN-γ gene, a CA repeat polymorphism in the first intron has five alleles; the common allele (allele 2, 24 bp long) correlates significantly with high levels of *in vitro* IFN-γ production (Pravica *et al*, 1999). Differential binding of nuclear factors has been reported at a polymorphism (+4766 C>T) in the 3′UTR (Bream *et al*, 2000). As yet, these have not been associated with pathologies such as cancer.

### Interleukins

Some Interleukins such as IL-8, IL-12, IL-10 and IL-4 influence tumour growth and angiogenesis by different mechanisms. Although several polymorphisms have been described in the encoding genes, only a few have been shown to be of functional importance. One promoter IL-10 polymorphism (−1082G>A) influences IL-10 production. The G allele (−1082G) is associated with higher cytokine production (Turner *et al*, 1997) and may increase cervical cancer risk (Stanczuk *et al*, 2001) and cutaneous malignant melanoma (Howell *et al*, 2001). At one of the IL-4 polymorphisms (−590C>T), the –590T allele is associated with increased promoter activity (Rosenwasser *et al*, 1995). This and other polymorphisms if proven to be of functional significance could represent potentially significant candidate genes in the regulation of tumour angiogenesis.

## STUDY DESIGN AND STATISTICAL CONSIDERATIONS

Genetic polymorphisms are being increasingly evaluated for their role in multifactorial conditions, including cancer, using population case–control studies. Such studies offer many advantages when compared to family studies including:

- Recruitment of large numbers of cases and controls.
- Detection of polymorphisms that confer relative risks as low as 1.5, which is not usually possible with family studies (Risch, 2000), thus allowing identification of low penetrance susceptibility loci.
- As cancers largely affect the middle and elderly age group, family studies like the transmission disequilibrium test and the affected sib pair analysis involving parents and sibs of patients are difficult to perform, as many will be deceased.

However, choosing an ideal control set for a population study is a difficult problem, as the age, sex and ethnicity of the case and control groups should be matched to enable appropriate conclusions to be made. Studies on functional gene polymorphisms will be more likely to yield positive results than random polymorphisms simply because of the greater prior probability of being associated with disease. However, polymorphisms in coding regions resulting in a non-conservative amino-acid substitution in conserved regions of the genome, or in potential transcription factor binding sites, are also studied because of their potential functionality. Other variants, even if not functional, can be associated with phenotype because of linkage to closely situated functional polymorphisms. It is now recognized that specific combinations of polymorphisms in a gene (haplotypes) might be of greater significance than individual polymorphisms, not only for a more efficient capture and analysis of common genetic variation (Johnson *et al*, 2001), but also from a functional view point (Daly and Day, 2001).

The numbers of patients and appropriately matched controls, needed to demonstrate a specific relative risk with adequate power and acceptable type I error risk in a case–control study would depend on the frequency of the polymorphism in the population. For example, to study a polymorphism with a rare allele frequency of 10% (expected to be associated with cancer with an odds ratio of 1.5) with a power of 80% and type I error rate of 0.05, 558 individuals would be required in each group. Studying rare polymorphisms (<5% rare allele frequency) requires thousands of patients to prove small associations (odds ratio of 1.5 or lower), which may be of little biological interest because of the rarity of the polymorphism in the general population. A detailed discussion of these and related issues can be found in several recent reviews (Daly and Day, 2001; Risch, 2000; Weinberg and Umbach, 2000).

## CONCLUSION

Angiogenesis is a multifactorial process regulated by a plethora of factors. Alteration in protein and/or receptor expression plays an important role in tumour angiogenesis and progression. Polymorphisms in the angiogenic genes/factors may in part explain the variation in tumour angiogenesis observed between individuals. The functional significance of polymorphisms can be determined by both *in vivo* studies and *in vitro* studies. Simultaneously, well-designed, large case–control studies are necessary to establish associations between polymorphisms and cancer, but as yet there are few such studies.

Individual polymorphisms, even if proven to be functional, may only contribute to (and not solely determine) the heritable variation in protein levels and/or function. Many protein molecules acting along different carcinogenic pathways influence the development and spread of tumours, and hence the final outcome. It is therefore possible that specific combinations of polymorphisms within one or several genes will have a greater impact on the final phenotype than the individual polymorphisms.

We have recently established a DNA repository containing samples of over 1800 breast cancer patients and controls; primarily to identify gene polymorphisms in angiogenesis-related genes that play an important role in tumour growth and progression. We have investigated SNPs in genes including TNF-α, VEGF and Endostatin for associations with breast cancer severity and susceptibility. Functional SNPs in the TNF-α promoter (−308G>A and –238G>A), in the 3′UTR of the VEGF gene (936C>T) (Balasubramanian *et al*, 2002) and in exon 42 of the Endostatin gene (G>A change) are not associated with breast cancer. However, the Endostatin polymorphism appears to predispose to breast tumour invasion (unpublished data).

Identification of the role of angiogenesis related gene polymorphisms in the pathogenesis of specific tumours would lead to an increased understanding of the disease process and potentially to risk stratification and prognostication. At the present time, polymorphisms in the VEGF, MMP and PA system and TNF genes seem to be promising in the quest for markers influencing the severity and extent of tumour angiogenesis. In parallel with the search for functional polymorphisms in angiogenesis related genes, epidemiological studies to detect associations of gene polymorphisms with disease phenotypes are desired.
